# A 0.049 mm^2^ 0.5-to-5.8 GHz LNA Achieving a Flat High Gain Based on an Active Inductor and Low Capacitive ESD Protection

**DOI:** 10.3390/mi16080852

**Published:** 2025-07-24

**Authors:** Dawei Dong, Zhenrong Li, You Quan, Xuanzhang He, Junyi Zhang, Chengzhi Li, Liyan Yu

**Affiliations:** School of Microelectronics, Xidian University, Xi’an 710126, China

**Keywords:** low noise amplifier, ESD, active inductor, noise, SiGe HBT

## Abstract

This paper introduces a 0.5–5.8 GHz low-noise amplifier (LNA) incorporating a gyrator-C-based active inductor (AI) and an enhanced deep trench isolation (DTI) electrostatic discharge (ESD) diode. Results suggest that AIs exhibit excellent consistency under various process voltage temperatures (PVTs) as well as input powers and the improved DTI diodes reduce parasitic capacitance by an average of 8.5% compared to conventional ones. In terms of circuit design, comprehensive analyses of gain flatness and noise are conducted. Fabricated using a 0.18 μm SiGe BiCMOS technology, the LNA delivers a high S21 of 18.3 ± 0.3 dB, a minimum noise figure of 2.6 dB, and an S11 and S22 of less than −10 dB over the entire frequency band. Operating from a 3.3 V supply voltage with a core area of 0.049 mm^2^, it consumes 10 mA of current.

## 1. Introduction

With the diversified development of wireless communication standards, the demand for multi-band compatibility in radio frequency (RF) front-end systems has become increasingly prominent [1]. Traditional multi-band reception schemes employing discrete low-noise amplifier (LNA) designs result in proportional increases in chip area and static power consumption [2,3]. Hence, compared with the discrete component scheme, chip integration technology achieves performance improvement and cost optimization of RF systems through a monolithic architecture.

The wideband LNA, as the core front-end component of ultra-wideband wireless receiver systems, must address design challenges including broadband impedance matching and noise-linearity trade-offs. Typical implementations employ cascode configurations and distributed amplification architectures, achieving bandwidth extension through active inductors and zero-pole compensation techniques [4,5]. However, parasitic *C*_ESD_ and *R*_ESD_ due to the structure of electrostatic discharge (ESD) protection devices can significantly disrupt the balance between these performances. Therefore, there is a significant necessity for a low parasitic ESD protection device to assist with the LNA design.

Inductors are critical components in Radio Frequency (RF) circuits, such as Inductor Capacitor Tank-Voltage Controlled Oscillators (LC-VCOs), LNAs, and filters. However, as the feature size keeps decreasing, spiral inductors no longer meet the trend towards being miniaturized and low-cost. Meanwhile, its inherent electromagnetic interference has become another major challenge for complex systems. Active inductors (AIs) have been intensively studied for their high quality as well as compact area in several recent works [6,7,8]. However, to increase the tunability of the AI, these jobs design the control bits off the chip. This complicates the external control scheme. To overcome this limitation, we propose an envelope detector along with an adaptive bias circuit to achieve on-chip control of the AI. In the following work, the AI is applied to the design of an ultra-wideband LNA.

## 2. Circuit Analysis

### 2.1. Active Inductor Design

The shunt resistor feedback topology achieves broadband matching through resistive feedback, demonstrating superior performance advantages over other broadband structures in the 0.5–5.8 GHz frequency range. Although this structure reduces circuit gain due to feedback and introduces thermal noise from the feedback resistor, its broadband matching capability makes it the final choice. In comparison, the bandpass filter-based topology, while achieving broadband matching by incorporating a bandpass filter, requires a large chip area for its input passive filter network. Additionally, the parasitic resistance of low-Q on-chip inductors degrades noise performance. The common-gate structure, despite its good linearity, low power consumption, and process voltage temperature (PVT) stability, suffers from poor noise performance. Even with transconductance enhancement techniques for optimization, it still cannot match the overall performance of the shunt resistor feedback topology in the 0.5–5.8 GHz range. Therefore, after comprehensively considering broadband matching, noise, and gain trade-offs, the shunt resistor feedback topology is ultimately selected as the implementation scheme for this design.

In the design of broadband low-noise amplifiers, traditional passive inductors struggle to achieve good impedance matching across wide frequency ranges due to their fixed inductance characteristics. The adaptive-biased active inductor employed in this study effectively addresses this issue through a dynamic tuning mechanism. By real-time adjustment of bias conditions, this structure enables the equivalent inductance to adaptively vary with frequency: providing higher equivalent inductance at low frequencies to improve input matching, while automatically reducing inductance at high frequencies to suppress parasitic effects. Therefore, the adaptive-biased inductor effectively extends the bandwidth of shunt-feedback broadband low-noise amplifiers.

**Figure 1:**
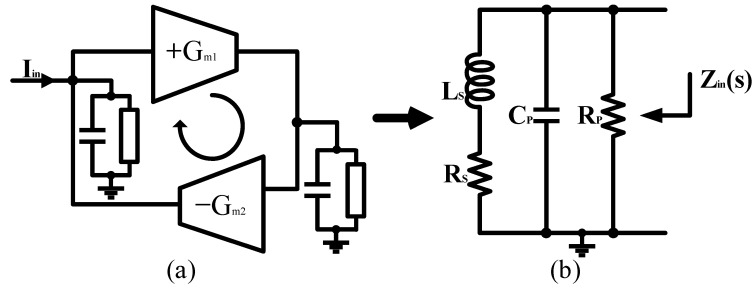
(**a**) The gyrator–C structure. (**b**) The lossy equivalent model for gyrator-C-based AI.

**Figure 2:**
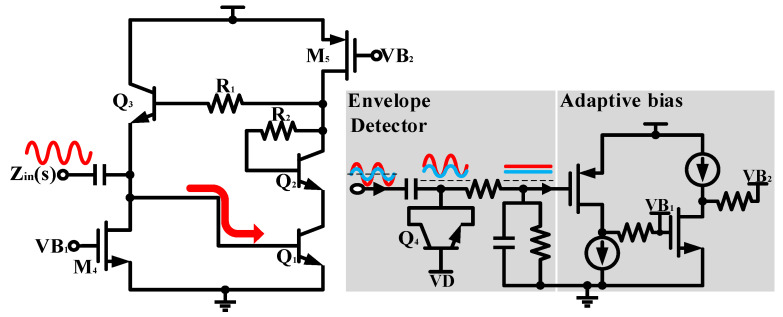
Schematic of the proposed active inductor and adaptive biasing circuit.

Depending on the state of the art, AIs can be configured in three ways: (1) amplifiers; (2) current conveyors; (3) gyrators [9,10,11]. The third family shows superiority in tunability, power consumption, frequency range, and circuit area. Figure 1a illustrates the gyrator–C structure. A gyrator-C consists of a positive trans-conductor and a negative trans-conductor connected head to tail. Considering the parasitics in the circuit, the gyrator-C-based AIs can be equated to the lossy model in Figure 1b. Figure 2 proposes the schematic diagram of the adopted gyrator-C based-Grounded AI. In this design, *Q*_1_ and *Q*_2_ form a cascode structure to provide high-output impedance and are used as a negative trans-conductance unit while the common-collector transistor *Q*_3_ is used as positive trans-conductance unit. MOSFETs *M*_4_ and *M*_5_ act as current sources to provide bias currents to the positive and negative trans-conductance unit, respectively. Resistor *R*_1_ biases *Q*_3_ on the feedback loop and increases the inductance of the Grounded AI. From the AC small-signal equivalent model, the port impedance can be reported as

(1) $$\frac{1}{Z_{in}\left(s\right)}=\frac{1}{r_{o4}}+sC_{\pi 4}+\frac{\left(1+g_{m2}r_{\vartheta })(sC_{\pi 5}+g_{m1}\right)}{1+sC_{\pi 5}\left(R_{1}+r_{\vartheta }\right)}$$

where $r_{\vartheta }=r_{o5}g_{m3}(r_{o4}//r_{\pi 3})$, $C_{\pi 4}{,\;C}_{\pi 5}$ are the equivalent capacitance between the base and emitter, *g_mx_* (x = 1,2,3…) is the trans-conductance, $r_{\pi x}$ (x = 1,2,3…) is the input impedance, and *r_ox_* (x = 1,2,3…) is the output impedance. The expression for inductance (*L*), series resistor (*R_s_*), parallel resistor (*R_p_*) and parallel capacitor (*C_p_*) in Figure 1b can be obtained from (1) as

(2) $$L_{S}=C_{\pi 5}\left(R_{1}/r_{\vartheta }+1\right)/\left(g_{m1}g_{m2}\right)$$

(3) $$R_{S}=1/\left(g_{m1}g_{m2}r_{\vartheta }\right)$$

(4) $$R_{P}=r_{o4}$$

(5) $$C_{P}=C_{\pi 4}$$

However, the instability of the AI at different PVT as well as input power raises risks for the circuit. The equivalent inductance (*L*_eq_) and quality factor (Q) of AIs are inherently dependent on transistor parameters such as transconductance and output impedance. PVT variations can cause significant drift in these parameters. Implementing adaptive biasing through dynamic adjustment of bias voltages/currents effectively compensates for PVT-induced variations, thereby maintaining AI stability. Furthermore, when handling large signals, the core transistors in AIs may enter nonlinear regions (cutoff or saturation), causing *L*_eq_ to vary with signal amplitude and introducing harmonic distortion. Adaptive Class-AB biasing that dynamically adjusts operating points based on input signal magnitude ensures transistors remain in their quasi-linear region, preserving AI linearity.

**Figure 3:**
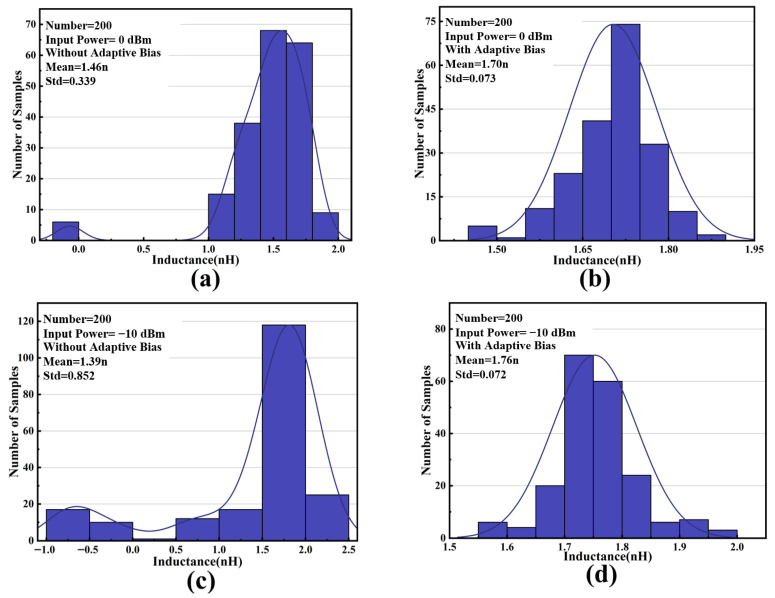
Monte Carlo simulation results for AI without adaptive bias under (**a**) 0 dBm and (**c**) −10 dBm and with adaptive bias under (**b**) 0 dBm and (**d**) −10 dBm.

Therefore, we use an envelope detector and an adaptive biasing circuit to achieve on-chip self-bias of the non-tunable AI. The different power input signals will be shaped into varying DC voltage levels through the envelope detector. Then, the adaptive bias circuit provides a dynamic static operating point for the AI to maintain its linearity. The base bias *V_D_* of diode-connected transistor *Q*_4_ is connected to a current mirror bias circuit to ensure full PVT coverage. Monte Carlo simulation results of the AI with and without the adaptive bias circuit at different input power are given in Figure 3. Obviously, the consistency of AIs is dramatically improved.

### 2.2. ESD Protection Devices

**Figure 4:**
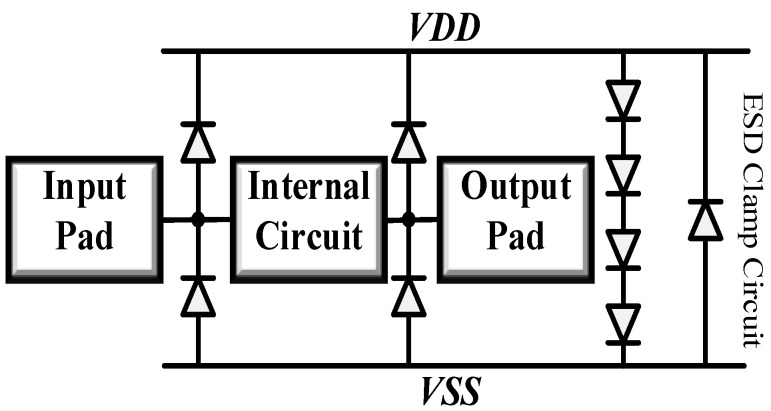
ESD protection circuit schematic diagram.

Figure 4 shows the ESD protection circuit schematic diagram. ESD diodes are widely employed in chip IO protection because of their simple structure. Nevertheless, there is a positive correlation between the robustness of the device and its parasitics. Specifically, a device with high robustness tends to generate more parasitics, while a device with fewer parasitics usually has lower robustness, as reported in references [12,13]. This issue poses a significant challenge for Radio Frequency Integrated Circuits (RFICs).

**Figure 5:**
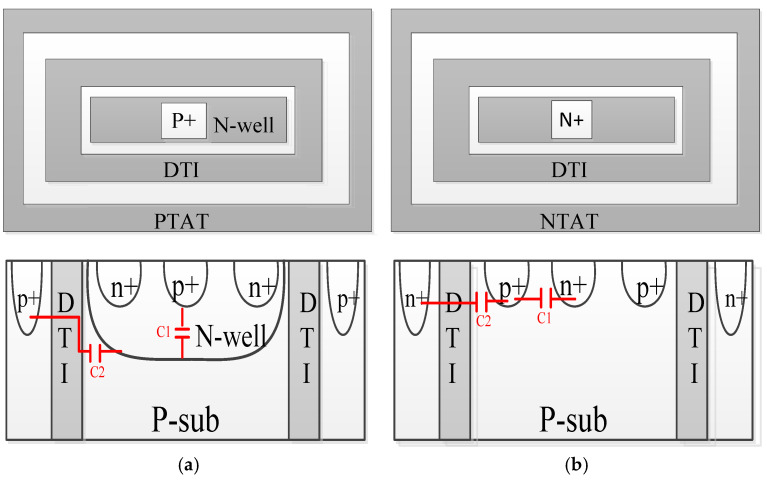
(**a**) P+/nwell DTI diode and (**b**) N+/psub DTI diode with DTI structure.

The schematic diagrams of the P+/nwell DTI diode and N+/psub DTI diode with the DTI structure are shown in Figure 5. The parasitic capacitance of each ESD diode includes the capacitance associated with the metal interconnect, the intrinsic capacitance from the ESD diode, and the parasitic pad capacitance [14,15,16]. This paper discusses only intrinsic capacitance. Take the P+/N-well diode (Dp) as an example. Parasitic capacitance consists of the capacitance *C*_1_ caused by the P+ diffusion/N-well junction and the capacitance *C*_2_ caused by the N-well/P+ guard ring junction. These capacitances can be considered as the barrier capacitance CT caused by the contact barriers between the P-type and N-type regions of the diode, which can be given by

(6) $$C_{T}(V)=A_{eff}\times {\left[\frac{\varepsilon_{0}\varepsilon_{\alpha }qN_{A}N_{D}}{2(N_{A}+N_{D})(V_{D}-V)}\right]}^{\frac{1}{2}}=A_{eff}\times C_{T}{\,}_{A0}(0)\times {\left(1-\frac{V}{V_{D}}\right)}^{-\frac{1}{2}}$$

where *A_eff_* is the effective area of the PN junction, *V*_D_ is the built-in potential. *C_TA_*_0_(0) represents the junction capacitance per unit area under zero bias conditions, which can be represented as

(7) $$C^{2}{{\,}_{T}}_{A0}(0)=\frac{\varepsilon_{0}\varepsilon_{\alpha }qN_{A}N_{D}}{2(N_{A}+N_{D})V_{D}}\propto \frac{1}{{\left(N_{A}\right)}^{-1}+{\left(N_{D}\right)}^{-1}}$$

This study effectively suppresses parasitic capacitance. The optimized design constructs a novel diode topology through Deep Trench Isolation (DTI) technology.

The deep trench isolation (DTI) structure penetrates beneath the N-well/P-substrate junction interface, employing oxide fillers with a reduced dielectric constant compared to silicon. This configuration significantly minimizes lateral capacitance at the N-well/P-substrate boundary through dielectric isolation. While maintaining the fundamental interdigitated electrode arrangement of standard diodes, the DTI-enhanced design achieves superior RF characteristics through improved electrical isolation and capacitive decoupling.

**Figure 6:**
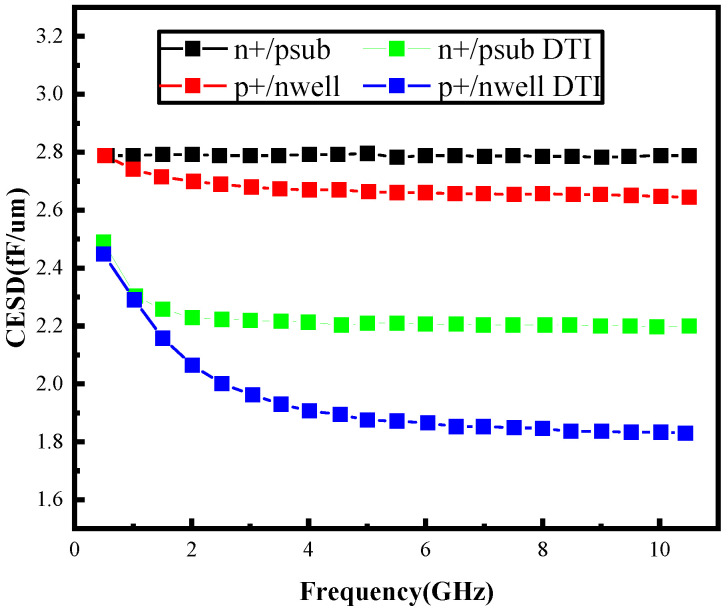
Simulation curve of parasitic capacitance.

To quantitatively evaluate this enhancement, four diode configurations were analyzed through Silvaco TCAD (Silvaco Inc., Santa Clara, CA, USA, https://silvaco.com/tcad/) simulations under identical dimensional parameters for comparative capacitance assessment. Figure 6 demonstrates a notable 8.5% average reduction in parasitic capacitance for DTI-incorporated ESD protection devices relative to conventional counterparts. This performance improvement primarily stems from the trench isolation’s effectiveness in suppressing stray capacitance between active components and peripheral guard ring structures. The experimental data confirms DTI technology’s capability to optimize high-frequency circuit performance while maintaining essential ESD protection functionality.

When optimizing the trade-off between parasitic capacitance and ESD protection capability, simulation results indicate that the maximum failure current of the DTI diode used in this design is 0.23 A/μm. Under the JEDEC (JEDEC Solid State Technology Association, Arlington, VA, USA) 2 kV HBM standard, the peak current reaches 1.35 A. Therefore, a minimum diode length of 5.87 μm is required to meet the 2 kV HBM ESD protection requirement. While increasing the ESD diode area can further enhance ESD robustness, it also introduces additional parasitic capacitance, which degrades circuit performance. Thus, a trade-off must be made between ESD protection capability and parasitic effects. Ultimately, this design adopts a 7 μm length, providing sufficient ESD protection margin while avoiding excessive parasitic capacitance.

**Figure 7:**
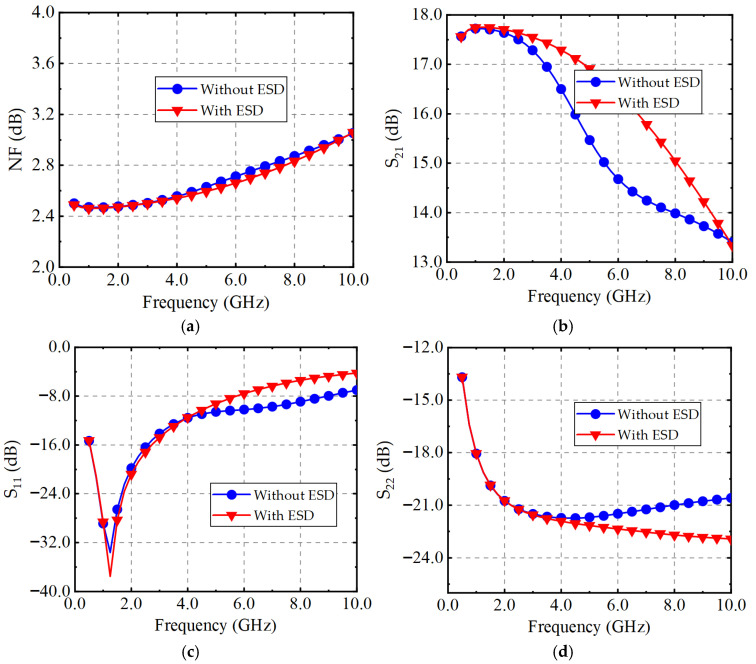
Simulation of (**a**) NF, (**b**) S21, (**c**) S11 and (**d**) NF for LNA with/without ESD protection circuit.

To evaluate the impact of the ESD protection circuit on LNA performance, simulations were conducted for LNA circuits with and without ESD. The simulation results are shown in Figure 7. Figure 7a,b present the simulation results for S11 and S22. The results indicate that the input and output matching of the overall circuit changes after integrating ESD devices. This is because the introduced diodes introduce parasitic capacitance, which acts in parallel with the input and output of the circuit, causing a shift in S11 and S22. To mitigate this shift, the parasitic capacitance should be reduced without compromising the ESD protection performance. Figure 7c shows the S21 simulation results. It can be observed that, since the matching design accounts for the influence of ESD, the LNA with ESD exhibits an advantage in gain, with a slower gain degradation at higher frequencies. However, at frequencies above 10 GHz, the gain of the LNA without ESD may exceed that of the proposed ESD-integrated LNA. This is due to the parasitic capacitance shifting the pole frequency downward, leading to a gain roll-off in the proposed LNA. Figure 7d displays the NF simulation results. After adding ESD protection devices, the noise figure of the circuit remains almost unchanged. Although the parasitic resistance of the diodes in the ESD protection circuit slightly degrades NF_min_, the ESD-integrated LNA achieves a lower NF due to adjustments in the input and output matching networks.

Since this design has not been fabricated yet, we acknowledge that actual silicon measurements for ESD performance (HBM 1 kV/2 kV or CDM 1 kV) are not available at this stage. Instead, we have estimated the ESD robustness based on the well-characterized protection levels of the original PDK ESD structures.

## 3. Circuit Design

**Figure 8:**
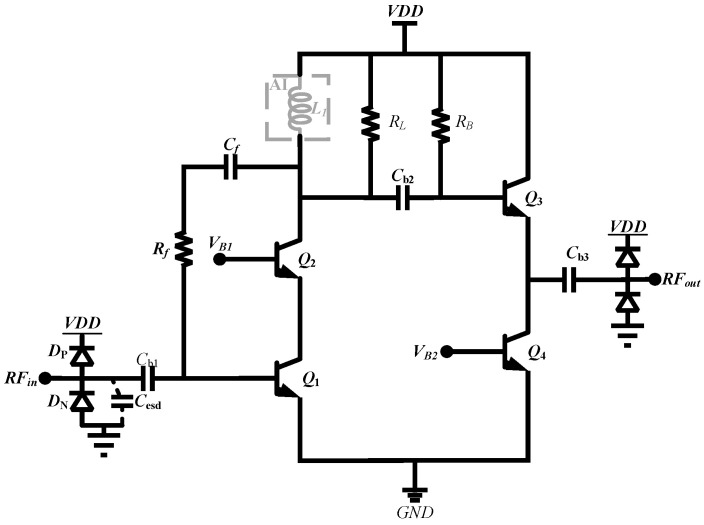
Schematic of the broadband cascode LNA with active inductor and low capacitive ESD protection.

Figure 8 shows the schematic diagram of the presented inductor-less wideband LNA. The load resistor *R_L_*, inductor *L*_1_ and the feedback resistor *R*_f_ extend the amplifier’s bandwidth. The transistors *Q*_1_ and *Q*_2_ provide sufficiently gain for the circuit, and *Q*_3_ and *Q*_4_ offer suitable output impedance. The capacitor CESD shows the parasitic capacitance of the ESD diodes, and *C*_b1_ is the blocking capacitor for coupling the RF signal to the base of Q1. Dp and N+/P-sub diode (Dn) are DTI diodes that provide a current discharge path for the circuit when ESD event occurs.

The electrostatic discharge (ESD) protection structure incorporates a cascode amplifier configuration to achieve optimal LNA performance while preserving enhanced ESD robustness. This design implementation employs DTI Dp and DTI Dn components featuring octagonal finger arrangements (8 fingers), with physical dimensions standardized at 31 mm × 16 mm. The respective parasitic capacitance measurements for these components are quantified as 62 fF and 78 fF, demonstrating controlled capacitive characteristics essential for high-frequency operation.

**Figure 9:**
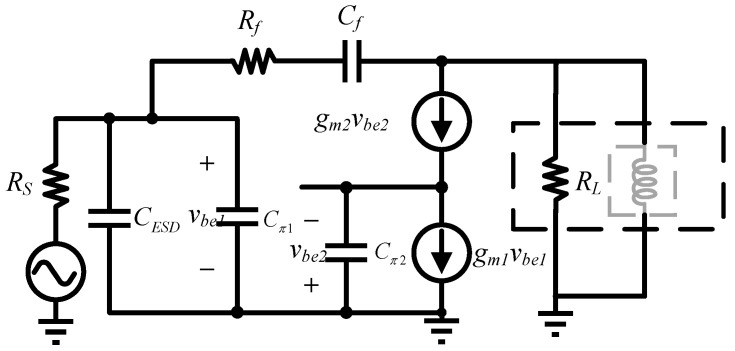
Schematic of the broadband cascode LNA with shunt resistive feedback.

A parallel resistive feedback network is implemented from *Q*_2_’s collector to *Q*_1_’s base to establish appropriate real impedance characteristics across broad frequency spectra. The analysis presented in Figure 9’s simplified small-signal model comprehensively evaluates input impedance behavior through circuit equivalent derivation; in particular, considering parasitic capacitance effects originating from ESD protection diodes and BJT device physics, the detailed input impedance can be derived as

(8) $$Z_{IN}=\frac{R_{f}+Z_{L}}{\left(1+g_{m1}Z_{L})+s(C_{\pi 1}+C_{ESD})(R_{f}+Z_{L}\right)}$$

(9) $$Z_{L}=R_{L}\left\|sL_{1}=\frac{sR_{L}L_{1}}{sL_{1}+R_{L}}\right.$$

**Figure 10:**
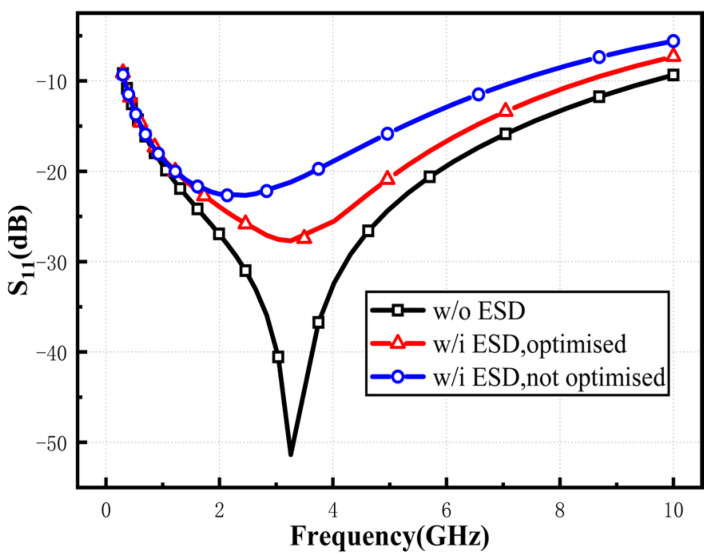
Simulated *S*_11_ with and without ESD.

Mismatch due to capacitance at high frequencies requires reducing the ratio of (*R_f_* + *Z_L_*)/(1 + *g_m_*_1_*Z_L_*) to satisfy constant impedance. Since *g_m_*_1_ and *Z_L_* are set for the static operating point, the mitigation effect of *R_f_* on the ESD parasitic capacitance is emphasized. Notably, there are tradeoffs to consider. Too large an *R_f_* will worsen bandwidth, while too small a value will degrade noise and gain. With a carefully chosen value of *R_f_*, the gain degradation will be within acceptable limits, and the bandwidth will be widened. In Figure 10, the impact of ESD circuitry on RF performance is significantly mitigated. If the same ESD is simply added without further circuit modification, a more prominent performance degradation occurs.

In RC networks where capacitors and resistors create a frequency pole, the signal gain decreases at a rate of −20 dB/decade. This characteristic necessitates maintaining frequency-independent gain stability across operational bandwidths to achieve optimal performance in broadband LNA. Numerous studies have extensively explored methodologies for broadband circuit implementations, as documented in prior research [17,18].

Taking into account the small signal circuit diagram shown in Figure 8, the voltage gain can be computed as

(10) $$|A_{V}|=\frac{g_{m1}g_{m2}R_{f}-g_{m2}-sC_{\pi 2}}{\left(1+\delta )(g_{m2}+sC_{\pi 2}\right)}$$

Here, δ = *R_f_/Z_L_*. To analyze the impact of gain flatness, we derive Equation (10). Ignoring the active inductor L_1_, we can obtain

(11) $$\frac{\partial A_{V}}{\partial s}=\frac{g_{m1}g_{m2}R_{f}C_{\pi 2}}{1+\delta }\cdot \frac{g_{m2}^{2}-\omega^{2}C_{\pi 2}^{2}-2sg_{m2}C_{\pi 2}}{{\left(g_{m2}^{2}+\omega^{2}C_{\pi 2}^{2}\right)}^{2}}$$

In Equation (11), the imaginary part is neglected, we can obtain

(12) $$|A_{V}^{\prime }|=\frac{g_{m1}g_{m2}C_{\pi 2}}{R_{f}^{-1}+R_{L}^{-1}}\cdot \frac{g_{m2}^{2}-\omega^{2}C_{\pi 2}^{2}}{{\left(g_{m2}^{2}+\omega^{2}C_{\pi 2}^{2}\right)}^{2}}$$

**Figure 11:**
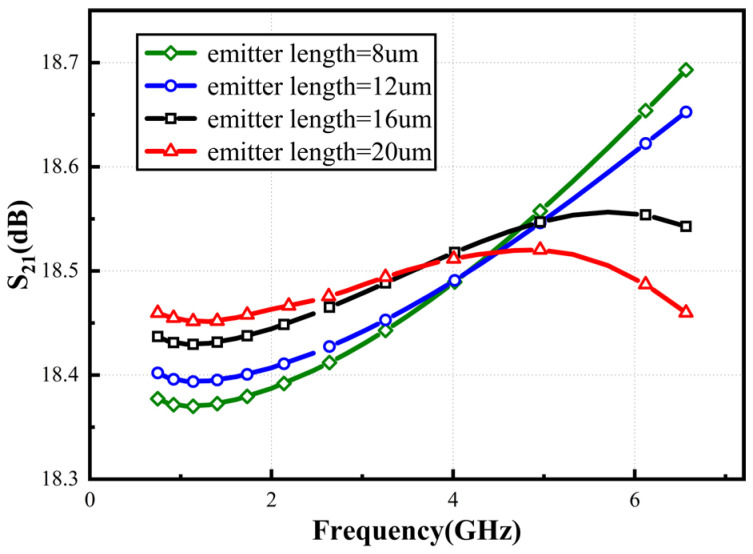
Simulated *S*_21_ with varied emitter length.

Equation (12) reveals that when $g_{m2}^{2}=\omega^{2}C_{\pi 2}^{2}$ the derivative of the small-signal gain with respect to frequency becomes 0. Under ideal conditions, with a transconductance *g_m2_* = 125 mS and an input capacitance *C_π2_* = 153 fF, theoretical calculations show that the gain derivative becomes zero at f = 130 GHz, achieving a flat gain response. However, in practical circuits, parasitic capacitances and resistances significantly reduce this characteristic frequency. Therefore, the actual frequency response must be evaluated through S21 parameter simulations. Without compromising other performance metrics, the flatness characteristic of the circuit gain can be enhanced by increasing the length of the *Q*_2_ emitter. Figure 11 shows the simulation curve of flatness as a function of emitter length. Specifically, increasing the upper transistor’s size optimizes the circuit’s flatness performance over the entire frequency band of interest.

**Figure 12:**
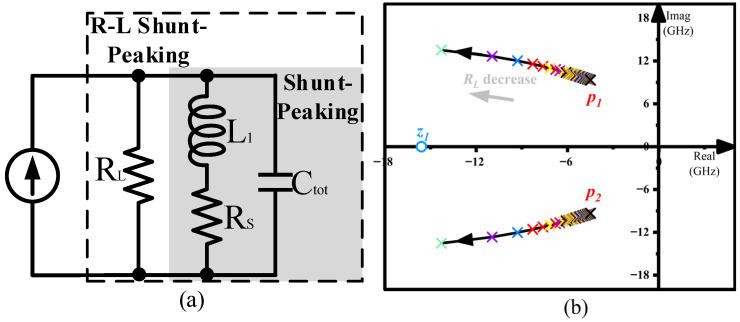
(**a**) A equivalent model of the R-L shunt-peaking technique. (**b**) Locus of poles and zeros as R_L_ decrease.

The flat gain performance of the circuit is achieved by adjusting the length of the emitter of *Q*_2_ in the absence of an inductor, and the bandwidth is further expanded by implementing the shunt-peaking technique with active inductors. Where the transistor is equated to a constant current source to explore the conditions for a load to achieve maximum bandwidth. The shunt-peaking technique has been proved to achieve up to 185% bandwidth expansion and 120% gain-peaking [19,20]. To solve the trade-off between bandwidth expansion and gain peaking, we connect a resistor RL in parallel with the original structure, resulting in the R-L shunt-peaking technique (Figure 12a), whose trans-impedance can be calculated as

(13) $$Z_{in}(s)=\frac{sL_{1}+R_{1}}{s^{2}L_{1}C_{tot}+s(R_{1}C_{tot}+L_{1}/R_{L})+1+R_{1}/R_{L})}$$

where *C_tot_* represents the parasitic capacitance of the output node. It can be found in Figure 12b that the impedance has a zero (z1) and a complex conjugate pair of poles (p1 and p2). Upon adding *R_L_*, z1 remains unchanged compared to the shunt-peaking technique, while p1 and p2 move away from the imaginary axis as RL decreases. Hence the magnitude of the frequency response decreases, while the position of the peak value of the frequency response (f = Im(p1)) is pushed back. The broadband characteristic of the load network is thus enhanced.

**Figure 13:**
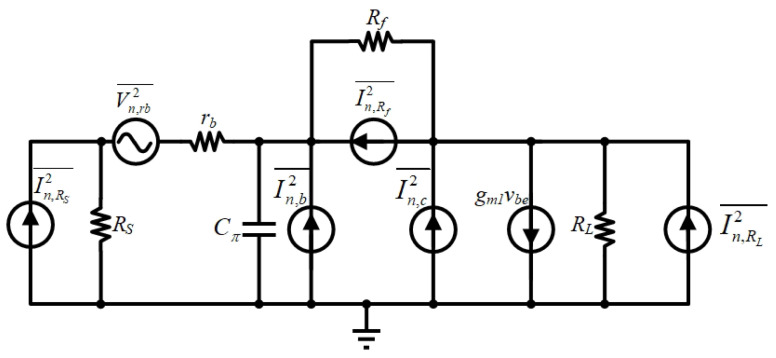
Small-signal noise model circuit.

For cascaded amplifiers, when the gain of the first stage is large enough, the noise figure is mainly related to the first stage. Due to the large output impedance of Q1, the noise of Q2 can be ignored. Figure 13 shows a simplified LNA equivalent noise model considering all significant noise sources.

(14) $$F_{rb}=\frac{r_{b}}{R_{s}}$$

(15) $$F_{nb+nc}=qI_{B}\cdot |Z_{S}//R_{F,in}{|}^{2}\frac{1}{2KTR_{S}}+\frac{qI_{C}}{g\prime_{m1}^{2}}\cdot |1+s(Z_{s}//R_{F,in})C_{\pi }{|}^{2}\cdot \frac{1}{2KTR_{S}}$$

(16) $$F_{R_{f}}=\frac{1}{R_{f}}\cdot \frac{1}{R_{S}}\cdot |Z_{S}//R_{F,in}{|}^{2}$$

(17) $$F_{R_{L}}=\frac{1}{R_{L}}\cdot \frac{1}{g\prime_{m1}^{2}}\cdot |1+s(Z_{S}//R_{F,in})C_{\pi }{|}^{2}\cdot \frac{1}{R_{S}}$$

(18) $$F_{r_{L1}}=\frac{1}{r_{L1}}\cdot \frac{1}{g\prime_{m1}^{2}}\cdot |1+s(Z_{S}//R_{F,in})C_{\pi }{|}^{2}\cdot \frac{1}{R_{S}}$$

where $Z_{S}=R_{S}//\frac{1}{sC_{ESD}}$, $R_{F,in}=\frac{R_{f}+R_{L}\left\|sL_{1}^{\prime }\left\|r_{L1}\right.\right.}{1+g_{m1}R_{L}R_{L}\left\|sL_{1}^{\prime }\left\|r_{L1}\right.\right.}$, *g′_m_*_1_ = *g_m_*_1_ − *R_f_^−^*^1^, and *F_rb_*, *F_Rf_*, *F_RL_*, *Fr_L_*_1_ and *F_nb+nc_* represent the noise factors due to thermal noise generated by the base parasitic resistor *r_b_*, the feedback resistor *R_f_*, the load resistance *R_L_*, the parasitic resistance r_L1_ and base and collector shot noise, respectively. The total noise figure can be calculated as

(19) $$\begin{array}{l} NF=1+F_{rb}+F_{R_{f}}+F_{R_{L}}+F_{nb+nc}+F_{rL1}\\ \;\;\;=1+\frac{r_{b}}{R_{S}}+\frac{|Z_{S}//R_{F,in}{|}^{2}}{R_{f}R_{S}}+\frac{\left|1+s(Z_{S}//R_{F,in})C_{ESD}{|}^{2}(2qI_{C}+4KT/R_{L}+4KT/r_{L1}\right)}{4KTR_{S}\times g{¢}_{m1}^{2}}\\ \;\;\;+\frac{qI_{B}|Z_{S}//R_{F,in}{|}^{2}}{2KTR_{S}} \end{array}$$

The noise generated by the base parasitic resistance *r_b_*_1_ of *Q*_1_ accounts for the most significant proportion, followed by the collector-emitter shot noise. As frequency increases, the influence of the collector-emitter shot noise gradually emerges, mainly thanks to the effect of the emitter junction capacitance *C_π_*_1_. By extending the emitter length and tuning the base bias voltage, the parasitic resistance at the base is diminished. Through co-optimization of emitter geometry scaling and bias conditioning, *r_b_*_1_ is simulated to reach 15 Ω and *C_π_*_1_ is simulated to be 153 fF, achieving an optimal trade-off between input return loss and voltage gain across the 0.5–5.8 GHz operational bandwidth.

## 4. Simulation Results

**Figure 14:**
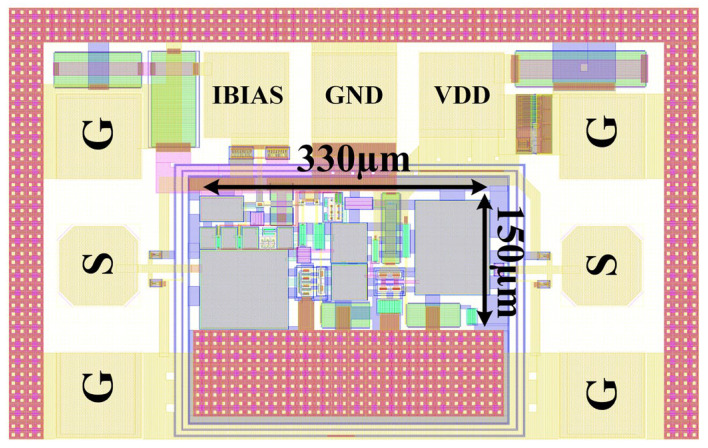
Layout of the proposed LNA.

The inductor-less wideband LNA described in this paper is fabricated using Tower-JAZZ’s 0.18 μm SiGe BiCMOS technology (Tower Semiconductor Ltd., Migdal Haemek, Israel). As shown in Figure 14, the core layout design of the LNA is highly compact, occupying a minimal chip area of only 0.049 mm^2^. Under a 3.3 V supply voltage, it exhibits an average current consumption of 10 mA, demonstrating efficient area utilization and optimized power consumption performance.

**Figure 15:**
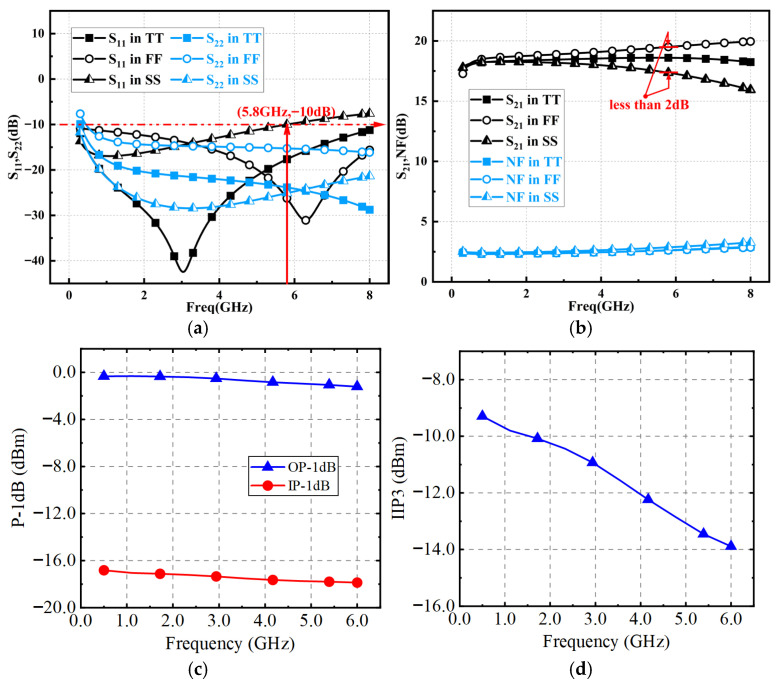
Simulated results of (**a**) S11 and S22 and (**b**) S21 and NF under different process corners and (**c**) P-1dB (**d**) IIP3 at TT corner.

Figure 15 gives the post simulation of LNA under different process corners. This paper simulates the circuit performance under different process corners of the transistor, including Typical N Typical P (TT), Fast N Fast P (FF), and Slow N Slow P (SS) corners. Figure 15a shows a good input and output matching is achieved. Figure 15b illustrates that the LNA achieves a peak S21 of 18.6 dB with a gain ripple below 0.6 dB. Meanwhile, the simulated NF spans from 2.6 to 2.78 dB across the operating bandwidth. Figure 15c,d shows the IP-1dB of −17 dBm, OP-1dB of −1 dBm and IIP3 from −14 dBm to −7 dBm. Table 1 summarizes the proposed LNA with some previously published LNAs. By applying the AI with adaptive bias circuit to the LNA, this circuit is competitive in terms of bandwidth as well as chip-area performance. While the layout has successfully passed DRC/LVS verification, minor discrepancies between actual measurements and simulation results may occur during practical implementation.

Table 1 compares the performance of the LNA proposed in this study with existing LNAs reported in the literature. The research findings demonstrate that this design achieves both excellent gain flatness and a low-noise figure. By eliminating the use of inductive components, the implementation has successfully minimized the chip area, thereby demonstrating significant competitive advantages in terms of area efficiency.

## 5. Conclusions

This paper explores the design and implementation of a broadband inductor-free LNA based on the Jazz 0.18 μm SiGe BiCMOS process, utilizing self-biased AI and enhanced DTI ESD diodes. The study analyzes the trade-offs and limitations in circuit design, proposes a resistive-inductive shunt-peaking technique, and conducts zero-pole analysis. Through the co-design of ESD diodes’ parasitic capacitance with the LNA circuit to optimize port matching, outstanding broadband performance is attained. The LNA core occupies a compact area of 0.049 mm^2^ and provides a flat high gain ranging from 18.0 to 18.6 dB across the 0.5–5.8 GHz frequency band.

## Figures and Tables

**Table 1:** Comparison between references and this work.

References	[21]	[22]	[23]	[24]	[25]	This Work
Tech	0.18 μm CMOS	0.18 μm CMOS	0.18 μm CMOS	0.18 μm CMOS	0.18 μm SiGe	0.18 μm SiGe
AI-based	NO	NO	YES	YES	NO	YES
Band Width (GHz)	0.1–6	0.1–2	1.8–2.6	0.05–10.8	8–20	0.5–5.8
Gain (dB)	15.5	17.5	11–12.57	10.6	14	18.0–18.6
NF (dB)	3–4	2.9–3.5	2.6–4.2	2.24	3	2.6–2.78
IIP3 (dBm)	1.5	7.5	−2	−2.3	−10	−9
Supply (V)	1.8	2.2	2	1.8	3.1	3.3
Current (mA)	8	9.68	5.7–6.2	3.4	15	10
Area (mm^2^)	0.4	0.63 *	0.0135	0.14 *	0.69	0.049
FoM **	9.62	4.05	1.1	29.88	6.51	12.36

* including pads. ** $\mathrm{FoM}=\frac{\mathrm{Gain}[\mathrm{lin}.]\cdot {\mathrm{BW}}_{3\text{-}\mathrm{dB}}\left[\mathrm{GHz}\right]}{(\mathrm{NF}[\mathrm{lin}.]-1)\cdot \mathrm{Power}[\mathrm{mW}]}.$

## Data Availability

The original contributions presented in this study are included in the article. Further inquiries can be directed to the corresponding author.
